# Gene–Environment Interactions in Repeat Expansion Diseases: Mechanisms of Environmentally Induced Repeat Instability

**DOI:** 10.3390/biomedicines11020515

**Published:** 2023-02-10

**Authors:** Stephanie Calluori, Rebecca Stark, Brandon L. Pearson

**Affiliations:** 1 Department of Environmental Health Sciences, Mailman School of Public Health Columbia University, New York, NY 10032, USA; 2 Barnard College of Columbia University, 3009 Broadway, New York, NY 10027, USA

**Keywords:** repeat instability, short tandem repeat, repeat expansion disease, gene–environment interactions, environment, oxidative stress, epigenetics

## Abstract

Short tandem repeats (STRs) are units of 1–6 base pairs that occur in tandem repetition to form a repeat tract. STRs exhibit repeat instability, which generates expansions or contractions of the repeat tract. Over 50 diseases, primarily affecting the central nervous system and muscles, are characterized by repeat instability. Longer repeat tracts are typically associated with earlier age of onset and increased disease severity. Environmental exposures are suspected to play a role in the pathogenesis of repeat expansion diseases. Here, we review the current knowledge of mechanisms of environmentally induced repeat instability in repeat expansion diseases. The current evidence demonstrates that environmental factors modulate repeat instability via DNA damage and induction of DNA repair pathways, with distinct mechanisms for repeat expansion and contraction. Of particular note, oxidative stress is a key mediator of environmentally induced repeat instability. The preliminary evidence suggests epigenetic modifications as potential mediators of environmentally induced repeat instability. Future research incorporating an array of environmental exposures, new human cohorts, and improved model systems, with a continued focus on cell-types, tissues, and critical windows, will aid in identifying mechanisms of environmentally induced repeat instability. Identifying environmental modulators of repeat instability and their mechanisms of action will inform preventions, therapies, and public health measures.

## 1. Introduction

Short tandem repeats (STRs), also known as microsatellites, are units of 1–6 base pairs that occur in tandem repetition to form a repeat tract [1]. STRs belong to the larger class of repetitive elements, which collectively comprise over 50% of the total human genome [2]. Specifically, STRs cover 1–3% of the human genome [3] and are located in coding and noncoding regions [4]. STRs are marked by their high susceptibility to mutation relative to non-repeat genome sequences. The mutation rate of STRs is approximately 10^−4^ to 10^−3^ per locus per generation, which exceeds the nucleotide substitution rate of 10^−8^ [5]. This gives rise to the phenomenon of repeat instability where mutations in STRs lead to expansions or contractions in the length of the repeat tract [4]. STRs and repeat instability play critical roles in contributing to organisms’ adaptation and evolution capabilities, as well as mediating health and disease [1,6].

Repeat expansion diseases are characterized by repeat instability and primarily affect the central nervous system and muscles. Approximately 50 diseases are currently classified as repeat expansion diseases, including Huntington’s disease (CAG repeat tract in *HTT* gene), Fragile X syndrome (CGG repeat tract in *FMR1*), myotonic dystrophy type 1 (CTG repeat tract in *DMPK*), Friedreich’s ataxia (GAA repeat tract in *FXN*), and frontotemporal dementia/amyotrophic lateral sclerosis (GGGGCC repeat tract in *C9ORF72*) [7]. Many repeat expansion diseases are progressive, becoming increasingly severe over time [8]. In such cases, the repeat tract length is often associated with the age of onset and disease severity. The longer the repeat tract, the earlier the age of onset and the more severe the disease [7]. Typically, repeat expansion diseases exhibit anticipation, the tendency of repeats to expand with each intergenerational transmission, which results in an earlier age of onset and may result in increased severity of symptoms [8,9]. Accordingly, repeat instability is a key determinant of disease pathology. Cancers [10,11,12,13,14], autism spectrum disorder [15,16,17,18], and psychiatric disorders [19,20,21,22] have also been found to involve repeat instability.

Gene–environment (G × E) interactions are suspected to play a role in the pathogenesis of repeat expansion diseases. Cigarette smoking, excessive physical activity, and dyslipidemia were identified as risk factors for amyotrophic lateral sclerosis (ALS) [23,24,25,26,27]. A recent Mendelian randomization study found that physical activity was correlated with altered gene expression, including *C9ORF72*, and was inversely proportional to age of onset among individuals with the *C9ORF72* expansion [28]. Such evidence has led researchers to postulate that G × E interactions contribute to ALS etiology [23,29]. In the case of Huntington’s disease, neonatal iron supplementation potentiated the disease phenotype in HD mice, indicated by the promotion of oxidative stress and energetic dysfunction in the brain. Effects of neonatal iron supplementation were not observed in wild-type mice, which suggests an interaction between the mutant huntingtin gene and iron [30]. Physical and cognitive activity, stress, and diet were also observed to modulate HD onset and progression in some clinical and animal model studies; however, evidence is limited and inconsistent [31,32]. Many environmental factors are associated with risk for autism spectrum disorder (ASD), including air pollution, heavy metals, herbicides, and pre- and post-conceptual drug use [33]. Since these environmental factors are known mutagens and genotoxicants, Pugsley et al. hypothesize that these toxicants play a role in de novo spontaneous mutations in ASD through three main mechanisms: directly altering DNA structure and inducing the DNA damage response (DDR), delaying or impairing the DDR, and indirectly inducing DNA damage through environmentally induced oxidative stress [33]. While emerging evidence indicates the presence of G × E interactions, how these interactions occur and contribute to pathology in repeat expansion diseases remain largely unexplored.

Here, we review the current state of knowledge on the mechanisms by which environmental factors modulate short tandem repeat instability in repeat expansion diseases. We focus on the interaction between environmental factors and repeat instability for several reasons. Short tandem repeats are highly unstable and susceptible to mutation, which may make them particularly vulnerable to environmentally induced mutagenesis [33]. In vitro and in vivo experiments have demonstrated the ability of environmental factors to promote repeat expansion and contraction (See entries (*) in Table 1). These studies were thoroughly reviewed elsewhere [8]. Furthermore, the molecular mechanisms of repeat instability, in the absence of exogeneous environmental factors, were actively investigated and reviewed elsewhere [9,34,35,36,37]. This foundation will facilitate our understanding of how environmental factors may contribute to or perturb mechanisms of repeat instability. Broadly, repeat instability may result from DNA repair pathways directly acting on repeat sequences or from impaired DNA repair responses and subsequent accrual of DNA damage. The specifics of repeat instability mechanisms are complex and varied. Repeat instability is influenced by many factors, including the sequence of the repeated unit, repeat tract length, the number of uninterrupted repeat units, the formation of secondary structures, cell type, and tissue type [8,9,38,39,40,41]. Repeat expansion and repeat contraction were also shown to occur by different mechanisms [9]. Finally, repeat instability is a key determinant of disease pathology, making it a prime therapeutic target. Identifying environmental modulators of repeat instability and their mechanism of action will aid in the creation of preventative and therapeutic strategies for repeat expansion diseases and inform public health measures.

We focused our literature search on the approximately 50 diseases currently classified as repeat expansion diseases [7]. First, we review the mechanisms by which environmental factors modulate short tandem repeat instability within adult somatic tissues, germline tissues, embryonic tissues, and cell reporter assays. It is critical to focus on *when* and *where* environmental factors are modulating repeat instability, because repeat instability is influenced by cell type and tissue type. For instance, it was shown that repeat instability occurs in both non-dividing (e.g., oocytes and postmitotic neurons) and dividing cells with distinct mechanisms for repeat expansion and contraction [42,43]. Genes activated as part of the DNA damage response also depend on cell type, stage of cell cycle, stage of organism development, and type of DNA [35,44]. This emphasizes the importance of focusing on *when* and *where* environmental factors are modulating repeat instability to accurately capture mechanisms. Furthermore, focusing on *when* and *where* environmental factors modulate repeat instability will aid in identifying targets for therapeutic and preventative applications in repeat expansion diseases. We also highlight the emerging role of epigenetics in modifying repeat instability and mediating the effects of environmental factors on repeat instability. Finally, future directions and challenges for research on environmentally induced repeat instability are discussed.

## 2. Environmental Factors Modulate Repeat Instability: Mechanistic Insights

### 2.1. The Link between Environmental Factors, Oxidative Stress, and Repeat Instability

When investigating environmentally induced repeat instability, researchers have primarily focused on environmental factors that induce oxidative stress. Oxidative stress occurs when there is an excess production of reactive oxygen species (ROS) and deficiency of the antioxidants needed to neutralize ROS [59,60,61]. This imbalance leads to oxidative damage in which ROS causes damage to DNA and other biomolecules, cells, and tissues [59]. DNA damage manifests as 7,8-dihydro-8-oxo-guanine (8-oxoG), other DNA adducts, and double- and single-strand breaks [59,62]. ROS include radicals, such as superoxide (O2^∙−^) and hydroxyl (OH^∙^), and nonradicals, such as hydrogen peroxide (H_2_O_2_). Typically, ROS are generated endogenously as by-products of oxygen metabolism [60]. Exogenous ROS production can also occur as a result of environmental exposure.

Many environmental factors (e.g., pollutants, heavy metals, certain drugs, cigarette smoke, radiation) are known to increase ROS production and cause oxidative stress [60]. Previous studies have identified oxidative stress as an important contributor to repeat instability. Oxidative stress can induce DNA damage, which may prompt DNA repair pathways that facilitate repeat expansion or contraction [33,37,48,63]. Oxidative stress was also implicated in the development and progression of cancers and neurodegenerative diseases, which often exhibit repeat instability [59,63]. This suggests that oxidative stress may serve as a link between environmental factors and repeat instability. The majority of studies reviewed below highlight the role of oxidative stressors and the induction of oxidative damage in repeat instability.

### 2.2. Adult Somatic Tissue: Environmental Factors and Repeat Instability

Through a series of in vitro experiments, Lai et al. [46] demonstrated the potential of oxidative stressors to promote repeat expansion via an MMR-BER hybrid mechanism, in which MSH2-MSH3 is essential for repeat expansion. Humans possess five major DNA repair pathways: base excision repair (BER), which corrects single-base lesions; nucleotide excision repair (NER), which corrects bulky DNA lesions; mismatch repair (MMR), which corrects errors in base-to-base alignment in replicated DNA; homologous recombination (HR), which targets double-strand breaks using template-dependent repair; and non-homologous end joining (NHEJ), which, in an error prone process, repairs double strand-breaks via ligation of two strands of damaged DNA [33,64]. Lai et al. specifically investigated components of BER and MMR, because they were previously implicated in promoting repeat expansion [65,66,67]. It is known that the BER pathway begins with a DNA glycosylase, such as OGG1, removing a damaged base, which forms an abasic site. The 5′-end is processed by apyrimidinic endonuclease 1 (APE1), which forms a gap that is typically filled by the DNA polymerase, polꞵ [46]. To assess crosstalk between MMR and BER, Lai et al. [46] used a synthetic BER template, consisting of a (GAA)_20_ or (CAG)_20_ repeat tract flanked on either side with 20 bases of random sequences, to mimic an abasic site after the removal of an oxidized base during base excision repair. Through in vitro experiments, it was shown that MSH2-MSH3 formed a physical complex with polꞵ at the APE1 nick site in the repeat tract. This finding was replicated in cultured human cells exposed to environmentally induced oxidative stress.

Human lymphoblast cells from an unaffected individual with a (GAA)_15_ repeat tract in the *FXN* gene were treated with DNA oxidizing agents potassium chromate (K_2_CrO_4_) or potassium bromate (KBrO_3_). Co-immunoprecipitation and co-localization experiments demonstrated that MSH2-MSH3 and polβ formed a direct physical complex, which increased upon exposure to the oxidative agents. ChIP assay confirmed that the MSH2-MSH3 complex formed only at the site of the GAA repeat tract in the *FXN* gene when exposed to the oxidative agents. This demonstrates that exposure to DNA oxidizing agents elicited a hybrid MMR-BER response at the site of the GAA repeat tract. Increased complex formation of MSH2-MSH3 and polβ was confirmed in untreated lymphoblasts from an individual with Friedreich’s ataxia (FRDA), harboring 280/830 GAA repeats [46]. However, exposure to the oxidizing agents did not increase complex formation, which was consistent with previous observations of already elevated levels of oxidative damage in lymphoblasts harboring disease-length alleles [46,68]. This may point to a similar phenomenon observed in CAG repeats in which cycles of DNA damage and repair contribute to changes in repeat length (expansions or contractions). However, once repeat length surpasses the disease-causing threshold, expanded repeats may impair the function of DNA repair proteins, leading to accrual of DNA damage and a toxic cycle of DNA damage/repair and repeat expansion [35].

To analyze the potential role of the MSH2-MSH3 and polβ complex in promoting repeat expansion, additional in vitro experiments were performed using the same synthetic BER templates of a (GAA)_20_ or (CAG)_20_ repeat tract [46]. In comparison to the BER pathway alone, the addition of MSH2-MSH3 suppressed repeat contraction and promoted repeat expansion. MSH2-MSH3 was found to promote repeat expansion by stimulating polβ to copy through the repeats rather than bypassing them. The presence of MSH2-MSH3 also stimulated strand displacement, allowing the strand to form a flap of increased size. The flap was largely protected from excision by FEN1, which allowed the flap to serve as the raw material for the repeat expansion. The results were independent of the (GAA)_20_ or (CAG)_20_ template used. These findings indicate that MSH2-MSH3 is essential for repeat expansion in response to oxidative damage. Collectively, these findings highlight the ability of exogenous oxidative agents to promote the formation of the MSH2-MSH3 and polβ complex in non-disease-length alleles, which has the potential to promote repeat expansion. This calls for future investigations into the potential role of exogeneous oxidative agents in facilitating the generation of disease-length alleles.

Based on their findings, Lai et al. [46] proposed an MMR-BER crosstalk model for repeat instability triggered by oxidative damage. Oxidative stress induces an oxidized DNA base in the repeat tract, such as 8-oxoG. The glycosylase OGG1 removes the 8-oxoG, leaving an abasic site, which is subsequently 5′-incised by APE1. In the absence of MSH2-MSH3, polβ opens the template and generates a single-strand DNA loop in the repeat tract. Polβ bypasses the loop as it cannot efficiently copy it. An endonuclease excises the loop, resulting in a repeat contraction. In the presence of MSH2-MSH3, the complex of MSH2-MSH3 and polβ loads onto the DNA at the APE1 incision site. MSH2-MSH3 stimulates polβ to copy through the repeat. Additionally, MSH2-MSH3 promotes strand displacement, which facilitates the formation of a flap that may take on a loop structure. MSH2-MSH3 inhibits FEN1 removal of the flap. However, MSH2-MSH3 becomes reoriented and allows flap-realignment on the damaged strand, which generates a new, shorter flap susceptible to FEN1 cleavage. An endonuclease then incorporates the residual nucleotides from the extended flap to generate the repeat expansion. Thus, Lai et al. present a potential model for how environmental factors could modulate repeat instability via the induction of oxidative damage and DNA repair pathways. Additional studies have provided support for the roles of MSH2 (MMR pathway) and OGG1 (BER pathway) in facilitating repeat expansion following oxidative damage.

H_2_O_2_ serves as an important model for oxidative stressors that may exacerbate repeat instability. Endogenous exposure to H_2_O_2_ occurs as it is generated by metabolic reactions. Excess H_2_O_2_ can damage the cell [59]. H_2_O_2_ can also generate OH^∙^, the most reactive of the free radical species [60]. Excess OH^∙^ can damage DNA, resulting in pyrimidine and purine adducts, DNA–protein cross links, and single- and double-strand breaks [59]. Exogenous exposure to H_2_O_2_ can occur as it is used in chemical industrial processes, for bleaching textiles and hair, and as disinfectants [69]. Kovtun et al. [47] demonstrated that acute exposure to H_2_O_2_ modulates somatic CAG repeat instability in Huntington’s disease, potentially through induction of oxidative damage and subsequent error-prone repair. Using human HD fibroblasts, Kovtun et al. [47] found that acute exposure to H_2_O_2_ induced CAG repeat expansion, resulting in medium-length and disease-length alleles. Treatment with H_2_O_2_ also resulted in a dose-dependent increase in single-strand breaks, which were repaired within two hours. Single-strand breaks may indicate BER pathway involvement. These results suggest that oxidative stressors may induce repeat expansion through error-prone repair of single-strand breaks, which may indicate BER pathway involvement.

Aging is a critical environmental factor to consider in the context of repeat instability, because the global population of older individuals is rapidly increasing. The global number of people aged 65+ is projected to triple from 2010 to 2050, reaching 1.5 billion people [70]. Aging is also associated with increased oxidative stress [60]. To examine the role of aging in environmentally induced repeat instability, Kovtun et al. [47] analyzed the level of oxidative damage to DNA using the marker 8-oxoG in the brain, liver, and tail of young (7–15-week-old) and old (15–52-week-old) R6/1 transgenic mice. The level of oxidation was similar between R6/1 and control mice of equivalent ages, indicating that the level of oxidation was dependent on aging and not the presence of the *HTT* transgene. The level and accumulation of age-dependent oxidative damage correlated with the degree of CAG repeat expansion. Liver and brain exhibited high levels of oxidative damage, and repeat expansion continued to progress with age in these tissues. CAG repeat expansions were confirmed in terminally differentiated neurons in the aging R6/1 mice when compared to those in the tail at 3 weeks. These findings suggest a potential relationship between age-dependent oxidative damage and repeat expansion, especially in the brain.

The association of age-dependent oxidative damage and repeat expansion was found to partly depend on the presence of OGG1, which is a DNA glycosylase in the BER pathway that primarily repairs 8-oxoG lesions [47]. R6/1/*OGG−*/*−* mice exhibited significantly suppressed or delayed age-dependent somatic CAG repeat expansion. In vitro, OGG1 was shown to participate in error-prone repair of DNA oxidative damage, initiating expansion through strand displacement/slippage during the gap-filling step of the BER pathway [47]. Kovtun et al. hypothesized a role for MSH2-MSH3 in this process; however, further investigation is required to validate if MSH2-MSH3 joins with the BER machinery to facilitate CAG repeat expansion in the context of age-dependent oxidative damage.

In addition to OGG1, Nei-like 1 (NEIL1) is a BER glycosylase that repairs a wider variety of oxidative lesions. The glycosylase activity of NEIL1 on the lesion 5-hydroxycytosine is essential for somatic and germline CAG repeat expansion in the R6/1 HD mouse model in a mechanism that may be independent of hairpin formation [67]. These findings suggest that NEIL1 functions in promoting repeat expansion in response to oxidative damage. Further research is needed to evaluate a role for exogenous oxidative agents in NEIL1-dependent repeat expansion.

In addition to investigating environmental factors that promote repeat expansion, researchers have examined environmental factors that mitigate repeat expansion. Since components of oxidative stress and the MMR pathway were implicated in repeat expansion, Gomes-Pereira and Monckton [58] evaluated the effects of chronic exposure to sublethal doses of cadmium (a reported inhibitor of MMR activity); the antioxidants melatonin, ascorbic acid, and Trolox C (molecules that work to neutralize ROS); and other compounds on repeat instability. Gomes-Pereira et al. previously established a mouse cell culture system with an unstable CAG•CTG repeat tract using a D2763Kc2 cell line derived from the kidney of a 6-month-old *Dmt-D* knockin mouse [57,71,72,73]. It was found that chronic exposure to manganese significantly reduced the rate of somatic repeat expansion, indicating a reduction of about 20% relative to untreated cells. In contrast, cadmium, melatonin, ascorbic acid, Trolox C, cobalt, zinc, H_2_O_2_, and ethanol did not significantly alter the repeat expansion rate. However, manganese and ascorbic acid were found to reduce repeat size variability, lowering somatic mosaicism in cell culture. Reduced repeat size variability may indicate changes to the frequency or magnitude of repeat expansion/contraction events. These results call for further investigation into the potential mechanistic role of manganese in reducing repeat expansion rate. Differences in dosage and exposure duration may account for the inconsistent findings from Kovtun et al. [47] with respect to the differential effect of H_2_O_2_ on somatic repeat expansion. Interestingly, cadmium and the selected antioxidants did not significantly alter the repeat expansion rate, although the antioxidant ascorbic acid reduced repeat size variability.

The modest effects of antioxidants on reducing repeat instability were also reported by Møllersen et al. [51]. R6/1 HD mice, harboring an approximately 115 CAG repeat tract, were given anthocyanin antioxidants from bilberry and blackcurrant in their drinking water daily from 4 to 22 weeks of age. The antioxidant treatment significantly reduced the repeat instability index in the ears and cortex compared to untreated R6/1 HD mice. However, Møllersen et al. noted that the effects of antioxidants were modest and did not significantly affect behavior in the R6/1 HD mice. Future studies are needed to investigate: (1) If antioxidants would be more effective at reducing or stabilizing repeat tracts if they were administered in combination with another potential modifier of repeat instability, (2) If antioxidants would be more effective if administered early, alone, or in combination with another repeat modifier as a preventative or therapeutic measure for those with normal, intermediate, or short disease allele lengths, and (3) If antioxidants would be more effective if administered at different dosages and durations.

### 2.3. Gametes and Germline Development: Environmental Factors and Repeat Instability

Numerous studies have uncovered the prevalence of parent-of-origin effects on intergenerational repeat instability in a range of repeat expansion diseases [74,75,76]. Parent-of-origin effects describe the phenomenon in which the sex of the affected parent influences the frequency and magnitude of repeat-length changes that occur during intergenerational transmission [74,75,76]. For instance, in Huntington’s disease, paternal transmission is associated with repeat expansion while maternal transmission is associated with repeat contraction [74]. In contrast, Fragile X syndrome (FXS) is largely associated with maternal germline instability [77]. Developmental windows and mechanisms of repeat instability in germ cells were reviewed elsewhere [78]. The consideration of parent-of-origin effects may help identify which parent(s) is at increased risk for environmentally induced repeat instability with respect to a given repeat expansion disease.

Potassium bromate (KBrO_3_), a DNA oxidizing agent, was shown to be mutagenic, cytotoxic, and carcinogenic [79]. KBrO_3_ is primarily used as an additive to flour. Many countries have partially or completely banned KBrO_3_ as a flour additive, but it is still permitted in the United States. KBrO_3_ is also used in hair products and dye for textiles, is a byproduct of the ozonation process sometimes used for disinfection of municipal drinking water, and may be used as an additive for fish paste, beer, and cheese [45,79]. Entezam et al. [45] demonstrated that KBrO_3_ exacerbates intergenerational repeat expansion in Fragile X syndrome through oxidative damage in a mouse model. Fragile X premutation mice [80], carrying ~130 CGG∙CCG repeats knocked in in the endogenous *Fmr1* gene, were raised on drinking water containing KBrO_3_ for at least two generations. Progeny from these exposed breeding pairs exhibited more repeat expansions compared to those from unexposed breeding pairs. Exposure to KBrO_3_ increased expansion frequency from 37% to 70% (1.9-fold) and from 62% to 83% (1.34-fold) in maternal and paternal transmissions, respectively. Exposure to KBrO_3_ had no significant effect on maternal or paternal contraction rates, indicating distinct mechanisms for repeat expansion and contraction. Paternal exposure resulted in larger repeat-length changes than maternal exposure. To gain insight into the mechanism by which KBrO_3_ exacerbates intergenerational repeat expansions, levels of oxidative damage were measured in maternally exposed mice. Levels of oxidative damage were not assessed in paternally exposed mice or in progeny. Elevated levels of 8-oxoG were found in the nuclei of oocytes from treated mice compared to untreated mice [45]. These findings suggest that KBrO_3_ increases the frequency of intergenerational repeat expansions in Fragile X via oxidative damage to DNA. However, these mechanistic findings should be evaluated in both maternal and paternal transmissions. Further experiments are needed to assess if specific pathways such as BER or MMR are involved in addressing the KBrO_3_-induced oxidative damage and facilitating repeat expansion in vivo.

### 2.4. Embryonic Development and Stem Cell Models: Environmental Factors and Repeat Instability

In addition to intergenerational repeat instability, repeat tract lengths observed in progeny are determined by repeat instability occurring throughout embryonic development. For instance, repeat instability during embryonic development was observed in Fragile X. In a Fragile X premutation mouse model, repeat expansions and contractions were observed in the early state of the two-cell embryo beyond the repeat expansions and contractions observed in ova [43]. MSH2 was found to play a role in facilitating repeat expansions during embryonic development [81,82]. Repeat instability was also observed to occur in pluripotent cells (e.g., human embryonic stem cells) and induced pluripotent stem cells (derived from human fibroblasts). Researchers have found evidence for oxidizing agents modulating repeat instability in stem cell models, which suggests a potential role for environmental factors modulating repeat instability in early embryonic development.

Jonson et al. [48] investigated the effect of H_2_O_2_ on CAG repeat instability in cultured HD murine embryonic stem cells (mESCs) derived from R6/1 mice harboring 127 CAG repeats. Chronic exposure to H_2_O_2_ promoted repeat expansion. CAG repeat tract lengths in treated HD mESCs significantly increased, averaging a threefold increase when compared to untreated HD mESCs. To assess potential mechanisms, Jonson et al. analyzed acute exposure to the same dose of H_2_O_2_. H_2_O_2_ was found to induce oxidative damage in the form of DNA double-strand breaks. Other forms of oxidative damage were not specifically evaluated. In response to H_2_O_2_ exposure, all selected DNA repair genes-*Msh2* and *Msh6* (MMR pathway), *Ku70* (NHEJ pathway), and *Neil1* and *Fen1* (BER pathway)-exhibited modest upregulation in HD mESCs compared to WT mESCs, except for *Ogg1*. The upregulation of the DNA repair genes *Msh2*, *Ku70*, and *Neil1* reached statistical significance. Further experimentation is required to investigate if altered gene expression has direct mechanistic implications for repeat instability. The significant upregulation of *Msh2*, *Ku70*, and *Neil1* may indicate a hybrid pathway for responding to the oxidative damage induced by H_2_O_2_ and potentially facilitating repeat instability.

Kovtun et al. [49] have provided support for the role of Msh2 in promoting repeat expansion in late embryonic development following exposure to H_2_O_2_. Repeated exposure to low doses of H_2_O_2_ resulted in repeat expansion when Msh2 was present in cultured mouse embryonic fibroblasts (MEF) isolated from *hHD*(*−*/*+*)/*Msh2*(*+*/*+*) mice at embryonic day 13. Further research is needed to assess if H_2_O_2_ exposure has similar effects on repeat instability in comparable human model systems. The potential for H_2_O_2_ to promote CAG repeat expansion in embryonic model systems should be tested in other CAG repeat expansion diseases in which Msh2 is found to promote repeat expansion [81,82].

In a follow-up experiment, Jonson et al. [48] chronically exposed cultured HD mESCs to KBrO_3_ or methyl methanesulfonate (MMS). Under these conditions, MMS, an alkylating agent, did not significantly alter repeat expansions. KBrO_3_, an oxidizing agent, generated a modest increase in repeat expansions and exhibited a significant increase in repeat expansions after the twelfth treatment, providing support for oxidative damage as a promoter of repeat expansion in HD mESCs.

The effect of environmentally induced oxidative stress on repeat expansion in stem cells was found to partly depend on the differentiation status of the cell. In addition to pluripotent HD mESCs, Jonson et al. [48] analyzed the effect of H_2_O_2_ on repeat expansion in HD mESCs undergoing differentiation. Treatment of actively differentiating HD mESCs with H_2_O_2_ resulted in significant and dose dependent increases in repeat expansion. In the absence of H_2_O_2_, the process of differentiation itself modestly induced repeat expansion. The effect of H_2_O_2_ treatment on differentiating cells was slightly lower than its effect on pluripotent cells, which suggests that pluripotent cells are especially vulnerable to environmental effects on repeat instability. The results of this study align with those from repeat instability studies in embryonic systems in the absence of environmental factors. In the absence of environmental factors, repeats exhibited stability or reduced instability after differentiation; repeat expansion was observed during pluripotency and in very early differentiation when cells were still actively differentiating [82,83]. It is unknown why repeat expansion appears to reduce after differentiation. One hypothesis is that differentiation-induced downregulation of RNA expression and protein levels of MSH2 consequently inhibit repeat expansion. The RNA expression and protein levels of MSH2 were observed to decrease in H_2_O_2_ treated HD mESCs and in untreated DM1 (myotonic dystrophy type 1) hESCs following differentiation [48,83]. The decrease in MSH2 correlates with a decrease in repeat expansions [83]. However, other studies have not reported a decrease in MMR proteins as a result of differentiation [82]. Further investigation is needed to evaluate how the differentiation state of the cell, alone or combined with environmental exposures, may influence repeat instability via a MSH2 dependent mechanism.

### 2.5. Reporter Cell Line Assays: Environmental Factors and Repeat Instability

Chatterjee et al. [52] demonstrated that acute exposure to cold, heat, hypoxia, or oxidative stress induced CAG repeat contraction via stress response pathways and DNA rereplication. A GFP-based assay was used to detect the effects of the environmental factors on repeat instability. The assay used HEK293 cells with a GFP(CAG)_89_ reporter and directly measured repeat contractions [84]. The occurrence of a repeat contraction would result in higher expression of the *GFP* gene. The results were replicated in a hypoxanthine-guanine phosphoribosyltransferase (HPRT) selection assay for CAG repeat instability in HT1080 cells [85]. Since the assays only measured repeat contractions, the findings of this study are limited to repeat contractions and cannot be applied to repeat expansions. Repeat expansion and contraction were previously shown to occur by different mechanisms [9].

Cells were acutely exposed to either cold, heat, hypoxia, or oxidative stress and were allowed to recover for 2–3 days. All four environmental factors significantly increased the frequency of GFP^+^ cells, as observed on recovery days 2 and 3, indicating a significant increase in the occurrence of repeat contraction. Sequencing was performed on a selection of GFP^+^ colonies isolated from cold, hypoxic, and oxidative stress conditions, which confirmed the induction of altered repeat tract lengths in the form of contractions or indels. In the case of contractions, the remaining repeat tracts ranged from 12–40 CAG units. Stress-response pathways were found to mediate the effects of the four environmental factors on repeat contraction. Knockdown of specific stress response factors largely reduced repeat contraction. The appearance of GFP^+^ cells was significantly decreased under cold exposure with CIRP knockdown; under heat with HSF1 knockdown; under hypoxia with HIF1, HIF3, NRF2, and HSF1 knockdown; and under oxidative stress with NRF2, HIF1, and HSF1 knockdown [52].

In addition, DNA rereplication was involved in the generation of the environmentally induced repeat contraction [52]. DNA rereplication is the erroneous firing of replication origins causing DNA to be replicated more than once in a cell cycle. When replicating DNA during mitosis, the DNA content in cells changes from 2C to 4C during S/G2 phases. A > 4 C-value for DNA content indicates additional rounds of replication, thus, signaling the occurrence of rereplication [52,86]. The percentage of cells with >4 C-value DNA increased from less than 5% in unexposed cells to more than 20% in cells exposed to each of the four environmental factors. The environmental stress-induced increase in the number of cells with >4 C-value DNA content and GFP^+^ cells was eliminated when CDT1, a factor involved in DNA rereplication, was knocked down [52,86]. Collectively, Chatterjee et al. demonstrate that stress response factors and DNA rereplication facilitate environmentally induced repeat contraction in cell assays.

The role of a replication-related mechanism in mediating environmentally induced repeat contraction was further supported by assessing other potential mechanisms. Transcription, MMR, and NER were not required for repeat contraction under conditions of environmental exposure in the cell assays [52,86]. Knockdown of OGG1 decreased occurrence of GFP^+^ cells in the case of exposure to oxidative stress, indicating the role of OGG1 (BER pathway) in mediating repeat contraction under conditions of oxidative stress in the cell assays [52]. This finding is consistent with Lai et al.’s model, in which the BER pathway promotes repeat contraction in the absence of MSH2-MSH3 when responding to oxidative damage [46]. Knockdown of replication factors FEN1 and PIF1 significantly reduced the occurrence of GFP^+^ cells across all four environmental factors, indicating support for the role of replication in mediating environmentally induced repeat contraction in the cell assays [52]. Together, these findings suggest that environmental factors induce repeat contraction in cell assays via stress response pathways and DNA rereplication, with the potential involvement of the BER pathway in the case of oxidative stress.

Chatterjee et al. [52] hypothesize that these pathways may impact normal cells under environmental stress or during development, or in abnormal cells as they evolve metastatic potential. Citing the documented role of DNA rereplication in genomic instability leading to cancer progression, Chatterjee et al. suggest that pathways for tumorigenesis and trinucleotide repeat instability intersect at trinucleotide repeats. Due to the role of repeat instability in numerous diseases, such as repeat expansion diseases, cancers, and psychiatric disorders, these findings have wide-ranging implications.

In a follow-up experiment, Chatterjee et al. [87] further investigated the mechanism by which the stress response and DNA rereplication generate repeat contraction in cell assay. Exposure to environmental factors was shown to induce contractions and indels in the GFP(CAG)_89_ assay [52]. Since indels are consequential markers of double-strand break repair, Chatterjee et al. investigated if double-strand break repair pathways process repeats during environmental exposure [52]. Two major double-strand break repair pathways exist: error-free homologous recombination (HR) and error-prone nonhomologous end joining (NHEJ). NHEJ consists of two pathways: classic NHEJ (c-NHEJ), which joins DNA ends together after minimal processing, and alternative NHEJ (alt-NHEJ), which depends on end resection to expose short sequence homologies. Chatterjee et al. demonstrated that alt-NHEJ is involved in mediating environmentally induced repeat contraction in cell assays. The HEK293(CAG)_89_ cells were acutely exposed to cold, heat, or hypoxic stress and allowed to recover for 3 days. The knockdown of key components in the alt-NHEJ pathway—PARP1, XRCC1, LIG3, and RAD50—significantly decreased the frequency of GFP^+^ mutants across environmental exposures, indicating a role for the alt-NHEJ pathway in facilitating environmentally induced repeat contraction. In contrast, knockdown of key components of homologous recombination and c-NHEJ had no effect on the frequency of GFP^+^ mutants under these environmental conditions. Chatterjee et al. note that it is possible that the double-strand breaks arise from the environmentally induced DNA rereplication, but this has yet to be investigated [87]. Collectively, these findings demonstrate that environmental factors induce repeat contraction in cell assays via cellular stress response machinery, DNA rereplication, and alt-NHEJ, likely through the creation and repair of double-strand breaks [52].

Further experimentation is required to assess if similar mechanisms of environmentally induced repeat contraction operate in other model organisms and systems. In addition, since the utilized assays were restricted to measuring repeat contraction, further research is needed to evaluate how this selection of environmental factors may promote repeat expansion and by what mechanisms. It is necessary to investigate if oxidative stress is involved in heat-, cold-, and hypoxia-induced repeat instability, because heat stress, cold stress, and hypoxia were found to stimulate ROS production [61,88,89]. Finally, since the rereplication mechanism was identified in cells replicating in culture, this selection of environmental factors may have different effects and operate via different mechanisms in vivo and in non-dividing cells.

## 3. Role of Epigenetics and the Environment in Modulating Repeat Instability

Epigenetic modifications were found to play a role in modulating repeat instability. CpG islands are primarily located in gene promoters and repetitive elements, including short tandem repeats [2], suggesting potential interactions between epigenetic modifications and repeat instability. Across a wide range of repeat expansion diseases, researchers have observed methylation differences between affected and non-affected individuals and have cited evidence for a relationship between methylation and repeat instability [90,91,92,93]. For instance, in SCA3, methylation levels in the *ATXN3* promoter were significantly higher in individuals with SCA3 when compared to unaffected individuals. Higher methylation levels were detected in individuals with earlier age of onset and families with an intergenerational CAG repeat instability [93]. Using bacterial and primate cell systems, Nichol and Pearson [94] demonstrated that methylation can significantly enhance or reduce repeat stability depending on the repeat sequence. In light of the associations between epigenetic modifications and repeat instability, researchers have started to elucidate the potential mechanistic interactions between them. Since environmental factors are known to induce epigenetic changes [95], investigations into the potential role of epigenetics as a mediator of environmentally induced repeat instability are needed.

### 3.1. The Role of Epigenetic Modifications in Modulating Repeat Instability

Several studies have found that the activity of histone deacetylase complexes (HDACs) and histone acetyltransferases (HATs) modulate repeat instability. Debacker et al. [96] demonstrated that the HDACs Rpd3L and Hda1 in yeast and the homolog HDAC3 in humans promote CTG repeat expansion. In both yeast and human cell models, inhibition of HDACs suppressed repeat expansion frequency, but did not affect repeat expansion size. Debacker et al. hypothesize that HDACs govern the initiation of expansion events. In addition, it was found that individual or combined knockdown of the histone acetyltransferases (HATs) CREB-binding protein and p300 increased repeat expansion frequency, suggesting that these HATs play a role in inhibiting repeat expansion or stabilizing repeats [96]. Similarly, House et al. [97] found that both acetylation by HATs and deacetylation by HDACs play a role in maintaining CAG stability, while changes in their dynamic regulatory activity may contribute to repeat instability.

Williams et al. [98] found HDAC3 to promote CAG repeat expansion in human tissue culture cells by directly interacting with Msh2-Msh3 (the MutSꞵ heterodimer of the MMR pathway). HDAC3 was shown to directly deacetylate five key lysine residues in Msh3. The deacetylation sites in Msh3 overlapped with a nuclear localization signal (NLS). Inhibition of HDAC3 resulted in partial relocation of MutSβ from the nucleus to the cytoplasm. This evidence provides support for HDAC3 deacetylating Msh3 to partly regulate the localization of MutSβ. Williams et al. propose a model in which import of MutSβ into the nucleus via the lysine-rich Msh3 NLS allows acetylation by p300/CBP and deacetylation by SMRT/HDAC3. Deacetylated MutSβ may then shuttle freely between the cytoplasm and the nucleus, allowing nuclear MutSβ access to triplet repeat DNA to promote expansions. Since HDAC3 inhibition prevented CAG repeat expansion without hindering canonical MMR activity, Williams et al. suggest that HDAC-selective inhibition may be a promising therapeutic approach.

In addition to HDACs and HATs, maintenance DNA methyltransferase (DNMT1), which preserves patterns of CpG methylation, was found to modulate repeat instability. Dion et al. [99] demonstrated that DNMT1 modulates CAG repeat instability in a human cell reporter assay and in the germline of a murine model of SCA1. Notably, their findings show that DNMT1 deficiency increases repeat expansion during intergenerational transmission in mice.

Collectively, these studies provide initial support for epigenetic modifications modulating repeat instability at specific repeat tracts, such as through changes in regulatory activity or through direct interactions with components of DNA repair pathways. Epigenetics might, therefore, mediate the relationship between environmental exposures and STRs.

### 3.2. Epigenetics as a Mediator of Environmentally Induced Repeat Instability

A few studies have evaluated the effects of 5-azacytidine, a DNA methyltransferase inhibitor, on repeat instability with varying results. Mollica et al. [50] demonstrated that chronic exposure to 5-azacytidine induces local hypomethylation and promotes CAG repeat stability in human HD fibroblasts. In contrast, Gorbunova et al. [56] demonstrated that chronic exposure to 5-aza-deoxycytidine promotes repeat instability in myotonic dystrophy and may induce genome-wide demethylation. An additional study by Gomes-Pereira and Monckton [57] found that chronic exposure to 5-aza-cytidine significantly reduced the repeat expansion rate in a murine cell model of myotonic dystrophy. These inconsistent findings could be due to differences in experimental design including differences in dosage, exposure duration, length of the repeat tract, the cell type, model organism, and the disease allele under study. In addition, Mollica et al. [50] studied local hypomethylation while Gorbunova et al. [56] focused on global hypomethylation induced by exposure. Differences in the scale of hypomethylation (local vs. global) and the specific genetic targets that were analyzed may account for the difference in observed effects on repeat instability. These studies only suggest a possible connection between the effects of 5-azacytidine on DNA methylation and its effects on repeat instability. Future research is needed to investigate if direct mechanistic links exist between environmentally induced changes in epigenetic modifications and repeat instability.

## 4. Future Directions

The field is beginning to identify the potential mechanisms by which environmental factors modulate short tandem repeat instability. Several strategies can be employed to gain further insights into these mechanisms.

A greater array of environmental factors should be explored with a focus on those most relevant to humans, such as pesticides, metals, air pollution, and radiation. Many environmental factors are known to induce oxidative stress [60], which is shown to be an important mediator of repeat instability. To more fully understand the effects of environmental factors on repeat instability, acute and chronic exposures should be tested using a range of sub-cytotoxic dosages across various timepoints.

The establishment of cohorts for repeat expansion diseases, collecting genetic and environmental data, can help guide research on the mechanisms of environmentally induced repeat instability [33]. Prospective cohorts monitoring individuals prior to disease onset could facilitate examining dynamic associations between environmental exposures and repeat length, as well as aid in the identification of critical windows and routes of exposure. Cross-sectional studies could also help identify single or multiple exposures associated with repeat length. Since many repeat expansion diseases are rare, establishing cohorts of sufficient size for robust statistical analyses may pose a challenge.

A wider variety of model systems is needed to better understand and validate the mechanisms of environmentally induced repeat instability. Animal models present limitations to the study of repeat instability. Rodents were observed to have different levels of sensitivity to some environmental factors and different degrees of repeat instability [56,100]. Mammalian model systems used to study CAG/CTG repeat instability, their limitations, and recommendations were reviewed elsewhere [37]. Some researchers are looking towards organoids to study repeat instability [37,101]. Human organoids present the opportunity to examine environmentally induced repeat instability in clinically relevant tissues, such as the brain and female germline, that are otherwise largely inaccessible. Recent advancements in 3D organoid technologies have generated models of female reproductive organs, including ovaries, fallopian tubes, and uterus, that can be used in toxicology studies and drug screens [102].

A potential challenge to progress in this field is a focus on a single peak allele metric, which may ignore potential cellular allele heterogeneity from bulk samples. The emergence of single-cell genomics and transcriptomics platforms represents an additional opportunity to query cell-type autonomous repeat instability. Additionally, the advent and growing predominance of long-read sequencing technologies represents an exciting advance to identify DNA repeats genome-wide and in targeted analyses (REF).

In addition to studying individuals affected by or at risk of repeat expansion diseases, it is necessary to study the potential effects of environmental factors on repeat instability in unaffected individuals. This is particularly salient in the case of germline repeat instability and intergenerational transmission. Jamali et al. [103] demonstrated that CAG repeat tracts of normal length in the *HTT* gene of sperm from healthy individuals undergo repeat instability during spermatogenesis. Among the 269 sperm analyzed from 3 men, approximately 3.3% underwent contraction or expansion. The existence of innate repeat instability in the human germline and its potential implications for disease risk and development in progeny underscore the importance of investigating the ways in which environmental factors may exacerbate or mitigate these processes. Doing so will aid in understanding how repeat tracts progress from normal-length to intermediate-length to disease-length alleles.

These strategies will aid future investigation into the mechanisms of environmentally induced repeat instability and will aid the creation of preventative and therapeutic strategies for repeat expansion diseases and inform public health measures.

## 5. Challenges

Our primary challenge is to understand how mechanisms of environmentally induced repeat instability intersect with mechanisms by which expanded repeats lead to cellular toxicity and pathology. It remains challenging to distinguish between environmental effects on repeat instability from secondary effects arising from the repeat itself. Depending on the repeat expansion disease, expanded repeats lead to cellular toxicity via loss of function of a protein or toxic gain of function on the RNA or protein levels [34]. In the case of HD, the expanded *HTT* gene encodes a mutant huntingtin (Htt) protein with an expanded polyglutamine domain. Expression of the mutant Htt was found to result in transcriptional dysregulation and alterations in epigenetic marks, such as DNA methylation and other histone modifications, that might be associated with and distinct from the pathological accumulation of poly-glutamine inclusions and cellular degeneration [104]. This may lead to an interactive loop between environmentally induced repeat instability, repeat expansions stimulating cellular toxicity, and cellular toxicity promoting repeat instability.

Similarly, a DNA damage/repair loop may present challenges to the study of environmentally induced repeat instability. The previous research on CAG repeat instability, in the absence of environmental factors, has found that DNA damage and repair contribute to changes in repeat length (expansions or contractions). However, once repeat length surpasses the disease-causing threshold, repeats are predisposed to further expansion. Many CAG repeats encode proteins with roles in DNA repair. Expanded repeats can impair the functions of these DNA repair proteins, which leads to a buildup of DNA damage and a continuous, toxic cycle of DNA damage/repair and repeat expansion [35]. If this model holds for environmentally induced repeat instability, it may be difficult to distinguish between environmental effects and the effects of the repeat itself.

## 6. Conclusions

**Figure 1:**
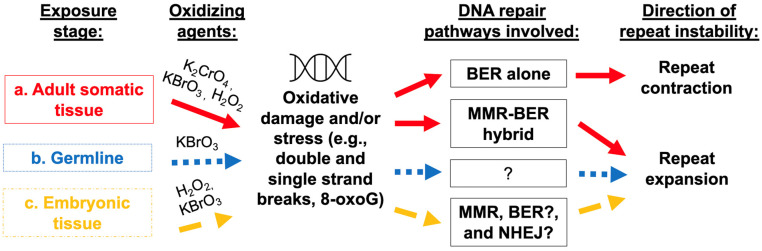
Environmental agents contribute to repeat instability. In adult somatic tissue (**a**), researchers have provided evidence for the role of an MMR-BER hybrid pathway in responding to potassium chromate or potassium bromate exposure, thus, leading to increased oxidative damage and susceptibility to repeat expansion. Additional studies support the role of components of BER and potentially MMR pathways in responding to oxidative damage, such as that induced by exogenous hydrogen peroxide or aging, again resulting in repeat expansion. Of note, when BER pathways alone are involved in responding to oxidative damage, evidence points to repeat contractions rather than expansions. Potassium bromate is shown to affect intergenerational repeat instability in germline cells via oxidative damage. The exact pathways that mediate this form of oxidative damage remain unknown; however, evidence suggests that oxidative damage in the germline (**b**) promotes repeat expansion. Differences in female and male germline instability call for future studies to investigate mechanistic differences in environmentally induced repeat instability in egg and sperm. Both hydrogen peroxide and potassium bromate exposure to embryonic tissue (**c**) is shown to promote repeat expansion. Studies have pointed to the role of MSH2 (component of MMR pathway), as well as suggest additional, potentially hybrid pathways, involving BER and NHEJ in mediating the response to oxidative damage and facilitating repeat expansion. Further research also showed that pluripotent cells are especially susceptible to repeat instability in response to environmental exposures.

Current evidence demonstrates that environmental factors modulate repeat instability via DNA damage and induction of DNA repair pathways, including MMR, BER, and alt-NHEJ, with differences in the mechanisms for repeat expansion and repeat contraction (summarized in Figure 1). Oxidative stress is shown to be a key mediator of environmentally induced repeat instability. Preliminary evidence suggests epigenetic modifications as potential mediators of environmentally induced repeat instability. Future research incorporating an array of environmental exposures, new human cohorts, and improved model systems, with a continued focus on cell-types, tissues, and critical windows will aid in identifying mechanisms of environmentally induced repeat instability. Short tandem repeat instability causes a wide range of repeat expansion diseases and typically influences age of onset and disease severity. Identifying environmental modulators of repeat instability and their mechanisms of action will inform preventions, therapies, and public health measures.

## Figures and Tables

**Table 1:** Effects of Environmental Factors on Repeat Instability. Several environmental factors have been found to modulate repeat instability across a variety of repeat expansion diseases and model systems.

References	Repeat Expansion Disease	Repeat Sequence	Model System	Environmental Factor	Exposure	Effect on Repeat Instability
[45]	Fragile X Syndrome	CGG•CCG	Fragile X premutation mice carrying ~130 CGG•CCG repeats in the endogenous *Fmr1* gene	KBrO_3_	0.5 g/L KBrO_3_ in drinking water; Mice raised on this drinking water for at least two generations	Parental exposure to KBrO_3_ increased expansion frequency from 37% to 70% and from 62% to 83% in maternal and paternal transmissions, respectively. Exposure to KBrO_3_ had no significant effect on maternal or paternal contraction rates. Paternal exposure resulted in larger repeat length changes than maternal exposure.
[46]	-	GAA	Human lymphoblast cell extracts from unaffected individual with (GAA)_15_ repeat tract in *FXN* gene	K_2_CrO_4_	Treated with 0.5 mM K_2_CrO_4_ for 2 h	MSH2-MSH3 complex only formed at GAA repeat tract in *FXN* gene after exposure to oxidative agents
				KBrO_3_	Treated with 10 mM KBrO_3_ for 2 h	
[47]	Huntington’s Disease	CAG	Human HD fibroblasts	H_2_O_2_	0.2 or 0.5 mM H_2_O_2_	Induced repeat expansion, resulting in medium-length and disease-length alleles
			Brain, liver, and tail of young (7–15-week-old) and old (15–52-week-old) R6/1 transgenic mice	Aging	-	Level and accumulation of age-dependent oxidative damage correlated with degree of repeat expansion. Liver and brain exhibited high levels of oxidative damage and expansion continued to progress with age.
[48]	Huntington’s Disease	CAG	HD murine embryonic stem cells (mESCs) with 127 CAG repeats; Derived from R6/1 mice	H_2_O_2_ ^a^	150 µM H_2_O_2_	Significantly increased repeat tract length, averaging a threefold increase when compared to untreated HD mESCs
				KBrO_3_ ^a^	7.5 mM KBrO_3_	Modest increase in repeat expansions and exhibited a significant increase in repeat expansions after the twelfth treatment
				Methyl methanesulfonate (MMS) ^a^	3 mM MMS	None
			Differentiating HD mESCs	H_2_O_2_	50 µM or 150 µM H_2_O_2_	Significant and dose dependent increases in repeat expansion; Effect of H_2_O_2_ on differentiating cells was slightly lower than its effect on pluripotent cells; Differentiation itself induced repeat expansion
[49]	Huntington’s Disease	CAG	Murine embryonic fibroblasts isolated from *hHD*(*−*/*+*)/*Msh2*(*+*/*+*) and *hHD*(*−*/*+*)/*Msh2*(*−*/*−*) mice at embryonic day 13	H_2_O_2_	Repeated exposure to low doses of H_2_O_2_	Resulted in repeat expansion when Msh2 was present
[50]	Huntington’s Disease	CAG	Human HD fibroblasts derived from individuals with HD; Mutant allele ranged from 42–60 repeats	5-azacytidine	10 µmol/L 5-azacytidine intermittently administered over 35 days	Stabilized the CAG repeat tract in HD cells while untreated HD cells exhibited small expansions
[51] *	Huntington’s Disease	CAG	R6/1 HD mice	Anthocyanin antioxidants from bilberry and blackcurrant	Anthocyanin antioxidants added to drinking water daily from 4–22 weeks of age	Reduced repeat instability index in ears and cortex; Effects were modest and did not significantly affect behavior
[52]	-	CAG	HEK293 cells with a GFP(CAG)_89_ reporter^e^	Cold	Cells incubated at 30 °C for 24 h	All four environmental stressors significantly increased the occurrence of repeat instability. Sequencing of independent GFP+ colonies isolated from cold, hypoxic, and H_2_O_2_ conditions showed substantially altered repeat tract lengths in the form of contractions or indels.
				Heat	Cells incubated at 44 °C for 24 h	
				Hypoxia	Cells incubated in 1% O_2_ in a hypoxic chamber for 48 h	
				H_2_O_2_	Cells treated with 0.5 mM H_2_O_2_ for 15 min	
[53] *	Myotonic Dystrophy type 1	CTG-CAG	Lymphoblastoid cell lines from unaffected individuals and individuals with DM1	Mitomycin C (MMC)	5 ng/mL MMC continuously administered for 12 population doublings	Enhanced expansion bias of long-pathogenic repeats and promoted expansion of normal-length repeats
[54] *	Myotonic Dystrophy type 1	CTG-CAG	Lymphoblast cell lines from individuals with DM1	Mitomycin C ^b^	0.1 or 0.2 µg/mL MMC for 14–16 h	Reduction of repeat length by 100–350 repeats often occurred
				Doxorubicin ^b^	0.1–5 µg/mL doxorubicin for 30 min	
				Ethylmethanesulfonate (EMS) ^b^	500 or 700 µg/mL EMS for 14–16 h	
				Mitoxantrone ^b^	50 or 100 nM mitoxantrone for 1 h	
[55] *	Myotonic Dystrophy type 1	CTG-CAG	Primary fibroblasts from 22.5-week female fetus with DM1 and 76-year-old woman with DM1	Aphidicolin ^c^	0.207 µM aphidicolin for a single population doubling	Enhanced the magnitude of repeat expansions
				Emetine ^c^	1 µM emetine for 18 h	
				Mimosine ^c^	200 µM mimosine for 18 h	None
[56]	Myotonic Dystrophy	CTG-CAG	Human fibroblast cell lines derived from individuals with myotonic dystrophy with (CAG)_80_ or (CAG)_150_ repeat tracts	5-aza-deoxycytidine	0.5 µM 5-aza-deoxycytidine	Induced repeat instability in the *DMPK* gene with a bias toward expansion
[57] *	Myotonic Dystrophy type 1	CAG-CTG	Dmt-D mouse kidney cell cultures	Caffeine ^d^	2 mM caffeine	High doses increased rate of expansion by ∼60%
				Novobiocin ^d^	60 µM novobiocin	None
				Aspirin ^d^	5.6 µM aspirin	Highly significant decreases in the rate of expansion ranging from ∼25% to 75%
				Cytosine arabinoside (AraC) ^d^	500 nM AraC	
				Ethidium bromide ^d^	250 nM ethidium bromide	
				H_2_O_2_^d^	100 µM H_2_O_2_	
				Rhodamine 6G ^d^	50 nM rhodamine 6G	
				5-azacytidine ^d^	10 µm 5-azacytidine	
[58] *	Myotonic Dystrophy type 1	CAG-CTG	D2763Kc2 cell line derived from kidney of 6-month-old *Dmt-D* knockin mouse	Manganese	2 µM each day for 73 days	Significantly reduced rate of somatic repeat expansion; Reduced repeat size variability
				Ascorbic acid	200 µM each day for 73 days	Did not significantly alter repeat expansion rate; Reduced repeat size variability
				Trolox C	500 µM in 0.1% ethanol each day for 73 days	Did not significantly alter repeat expansion rate
				Melatonin	20 µM in 0.1% ethanol each day for 73 days	
				Cadmium	2 µM each day for 73 days	
				H_2_O_2_	20 µM each day for 73 days	
				Cobalt	2 µM each day for 73 days	
				Zinc	2 µM each day for 73 days	
				Ethanol	0.1% each day for 73 days	

* These studies only observed treatment effects of environmental exposures on repeat instability. The possible mechanisms of environmentally induced repeat instability were not investigated. ^a^ Cells were exposed to their respective treatment once per passage for 12 passages. ^b^ Cell cultures were grown for up to 10–30 generations. In select experiments, a second dose of the drug was administered after recovery of the culture. ^c^ Cells underwent five rounds of their respective treatment. ^d^ Treated cultures were given fresh drug-supplemented medium every 2–3 days over the course of 3 months.

## Data Availability

No new data were generated.
